# Satellites for long-term monitoring of inland U.S. lakes: The MERIS time series and application for chlorophyll-a

**DOI:** 10.1016/j.rse.2021.112685

**Published:** 2021-12-01

**Authors:** Bridget N. Seegers, P. Jeremy Werdell, Ryan A. Vandermeulen, Wilson Salls, Richard P. Stumpf, Blake A. Schaeffer, Tommy J. Owens, Sean W. Bailey, Joel P. Scott, Keith A. Loftin

**Affiliations:** a NASA Goddard Space Flight Center, Ocean Ecology Laboratory, Greenbelt, MD 20771, USA; b Universities Space Research Association (USRA), Columbia, MD 21046, USA; c Science Systems and Applications Inc., Lanham, MD 20706, USA; d U.S. Environmental Protection Agency, Office of Research and Development, Durham, NC 27711, USA; e NOAA, National Ocean Service, Silver Spring, MD 20910,USA; f Science Application International Corp., Reston, VA 20190, USA; g U.S. Geological Survey, Kansas Water Science Center, Lawrence, KS 66049, USA

**Keywords:** MERIS timeseries, Inland waters, Remote sensing, Algorithm validation, Chlorophylla, Water quality

## Abstract

Lakes and other surface fresh waterbodies provide drinking water, recreational and economic opportunities, food, and other critical support for humans, aquatic life, and ecosystem health. Lakes are also productive ecosystems that provide habitats and influence global cycles. Chlorophyll concentration provides a common metric of water quality, and is frequently used as a proxy for lake trophic state. Here, we document the generation and distribution of the complete MEdium Resolution Imaging Spectrometer (MERIS; Appendix A provides a complete list of abbreviations) radiometric time series for over 2300 satellite resolvable inland bodies of water across the contiguous United States (CONUS) and more than 5,000 in Alaska. This contribution greatly increases the ease of use of satellite remote sensing data for inland water quality monitoring, as well as highlights new horizons in inland water remote sensing algorithm development. We evaluate the performance of satellite remote sensing Cyanobacteria Index (CI)-based chlorophyll *a*lgorithms, the retrievals for which provide surrogate estimates of phytoplankton concentrations in cyanobacteria dominated lakes. Our analysis quantifies the algorithms’ abilities to assess lake trophic state across the CONUS. As a case study, we apply a bootstrapping approach to derive a new CI-to-chlorophyll relationship, Chl_BS_, which performs relatively well with a multiplicative bias of 1.11 (11%) and mean absolute error of 1.60 (60%). While the primary contribution of this work is the distribution of the MERIS radiometric timeseries, we provide this case study as a roadmap for future stakeholders’ algorithm development activities, as well as a tool to assess the strengths and weaknesses of applying a single algorithm across CONUS.

## 1. Introduction

Lakes and other inland waterbodies cover ∼3% of the Earth’s continental surface (Downing et al., 2006). They provide critical ecosystems and habitats and offer essential support for human health and well-being by providing drinking water, food, and recreation. Furthermore, lakes contribute to global-scale processes through their influence on many aspects of the biosphere, including methane fluxes (*e.g.*, Bastviken et al., 2004; Walter et al., 2006), carbon cycles (MacKay et al., 2009; Mendonça et al., 2017), and other factors that regulate Earth’s climate (*e.g.* Cole et al., 2007; Downing, 2009, 2010; Raymond et al., 2013; Tranvik et al., 2009). Global lake conditions are often sensitive to human behaviors, such as land use changes, and thus may benefit from management and mitigation activities. It is therefore beneficial that lakes are monitored for long-term changes in water quality and anthropogenic induced responses that can influence human and ecosystem health, as well as global-scale processes. Robust and consistent water quality data sets remain critical for effective and interpretable lake monitoring.

Measurements of near-surface concentrations of the photosynthetic pigment chlorophyll-a (*Chl*; μg L^−1^) provide a useful and commonly measured metric of water quality. Most federal, state, and local *in situ* water quality monitoring programs collect *Chl*, albeit with varied measurement techniques (U.S. Environmental Protection Agency, 2000, 2009). The most conventional approach to obtaining *Chl* is collecting a discrete *in situ* sample from a dock or boat/ship (U.S. Environmental Protection Agency, 2011). Although high quality *in situ*-based sampling provides an essential way to measure a comprehensive suite of lake conditions and variables, it has limitations, especially in terms of temporal and spatial data set coverages. The large number of inland waters, many of which are in remote locations, makes routine *in situ* sampling costly and logistically challenging, if not nearly impossible over long distances and durations (Papenfus et al., 2020). Single or limited samples from a waterbody may not provide meaningful synopses of the state of the entire waterbody (*e.g.*, Lesht et al., 2018; Kallio et al., 2003; Vos et al., 2003) While temporal limitations can be partially overcome using continuous probes, their spatial distributions remain sparse.

Satellite-borne ocean color instruments offer useful complements to *in situ* data collection that overcome spatio-temporal sampling limitations. At a minimum, these spectroradiometers measure visible and near-infrared (NIR) radiances at discrete wavelengths at the top-of-the atmosphere. Broadly speaking, atmospheric correction algorithms are applied to remove contributions of the atmosphere and surface reflection from the total signal (*e.g.*, Mobley et al., 2016). Bio-optical algorithms are then applied to the remaining aquatic reflectances to produce estimates of biogeophysical properties, such as *Chl* (*e.g.*, Hu et al., 2012, 2019; O’Reilly and Werdell, 2019), near-surface concentrations of suspended sediments (*e.g.*, Ondrusek et al., 2012), spectral inherent optical properties (Werdell et al., 2018), and other indices of water quality and composition (*e.g.*, Lee et al., 2007; Olmanson et al., 2008). Such remotely sensed data records can be used effectively to assess long-term system changes on broad spatial and temporal scales, as well as observe dynamic short-term events, such as episodic algal blooms. These data records can also be reevaluated retrospectively as remote sensing methods improve and lake management and mitigation needs evolve. Altogether, these capabilities offer an increased ability to quantify satellite resolvable inland water bodies’ ecosystem events, such as harmful algal blooms, enabling statistical inferences on their temporal frequency, spatial extent, magnitude, and occurrence (Clark et al., 2017; Urquhart et al., 2017; Mishra et al., 2019; Coffer et al., 2020).

**Fig. 1.**
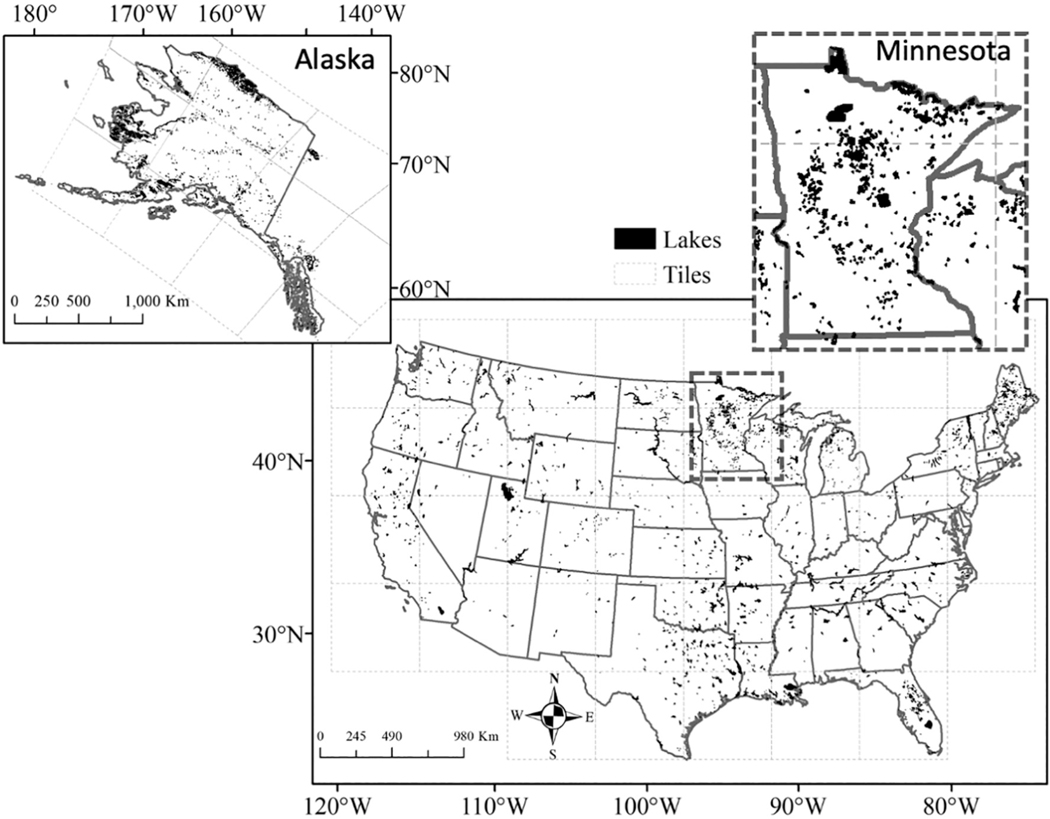
Maps of resolvable lakes for CONUS and Alaska. Black represents a satellite resolvable lakes for inland waters data set. A Minnesota inset map (dashed grey rectangle) highlights the large number of lakes across the state, which account for 70% of the lakes in this study. Resolvable lakes in CONUS meet a minimum three satellite pixel requirement. Alaska lakes only have a single pixel requirement; therefore, smaller lakes are shown in Alaska.

Satellite data sets provide value for early warning event detection and decision support when other monitoring resources are limited or not available for deployment. Additionally, annual potential avoided costs associated with satellite *Chl* measures were estimated between $5.7 and $316 million US$ (Papenfus et al., 2020). That said, in practice they provide a complement to, not a replacement for, *in situ* lake monitoring, as remote sensing measurements have intrinsic challenges. First, the temporal distributions of useful retrievals are dictated by the confounding interferences of clouds and atmospheric aerosols (Frouin et al., 2019) and satellite-specific orbital repeatability (IOCCG, 2007), both of which influence the frequency with which any given water body is observed. Second, ocean color measurements are restricted by nature to a spectrally-dependent near-surface layer (Gordon and McCluney, 1975). Third, the measurements are considered most reliable when collected offshore because of complications with adjacency effects (Bulgarelli et al., 2014) and mixed land-water pixels, both of which limit the assembly of a complete synopsis of a waterbody. Additionally, most satellite instruments designed for global ocean color have ground sample sizes (*e.g.* sensor pixel size) between 300 and 1000 m (Werdell and McClain, 2019), which limit the number of resolvable water bodies (Clark et al., 2017). As such, the inland waters discussed in this paper refer primarily to larger lakes and reservoirs and not narrower or smaller waters such as streams, rivers, smaller lakes/reservoirs, and ponds (Fig. 1). The ground sample size also establishes a lower limit on the spatial scale of any aquatic feature that can be detected (that is, intrapixel variability cannot be resolved). Finally, ocean color remote sensing provides a finite number of available biogeophysical products relative to the larger number of variables that can be measured *via in situ* sampling (*e.g.*, nutrient and toxin concentrations cannot be directly estimated from ocean color alone) (IOCCG, 2018).

Despite some limitations, 40 years of satellite ocean color has successfully facilitated insights into the long-term state of waterbodies, as well as their spatial and temporal variabilities (*e.g.*, Binding et al., 2015; Dutkiewicz et al., 2019; Gregg et al., 2017). Heritage satellite ocean color algorithms, however, were developed primarily for the global ocean and cannot always be appropriately applied to lakes and reservoirs given their optical complexity, overlying atmospheric conditions and altitudes, and proximity to land, to name only a few confounding issues (IOCCG, 2018). A variety of approaches has more recently emerged to address these challenges and extend *Chl* estimates from the oligotrophic open ocean to diverse systems and hypereutrophic lakes. Neil et al. (2019) presents a thorough review of 19 different approaches to derive inland water *Chl* from satellite ocean color that consider a range of empirical, semi-analytical, peak height methods, and neural network methods and an assessment of their performance in optically complex inland waters. Their results revealed the difficulty of establishing a single standardized approach. A need exists, however, to standardize metrics used for water quality assessment, especially for management that has human health implications (Clark et al., 2017; Coffer et al., 2020; Mishra et al., 2019; Urquhart et al., 2017). Standardized remote sensing products would also provide a foundation with which to compare lake quality assessments around the planet, which would ultimately reduce uncertainties related to their consideration in comparative or diagnostic models. Furthermore, meaningful scientific insights, such as trend analyses, can only be robustly realized with standardized data processing applied to the extended satellite record. To that end, a well-vetted, publicly available satellite inland water data record would greatly reduce the efforts associated with algorithm development and performance assessment, while also providing a consistent data set to enable broad algorithm refinement as well as determination if an approach requires regional adjustments.

The Cyanobacteria Assessment Network (CyAN) (Schaeffer et al., 2015) is one effort to apply consistent remote sensing methods to operational monitoring of inland water bodies, primarily lakes and reservoirs. CyAN is a joint U.S. Environmental Protection Agency (EPA), National Aeronautics and Space Administration (NASA), National Oceanic and Atmospheric Administration (NOAA), and U.S. Geological Survey (USGS) effort with a goal to produce and distribute operational, low latency remotely sensed metrics of cyanobacteria dominated harmful algal blooms (cyanoHABs) presence across observable contiguous United States (CONUS) and Alaskan lakes. The European Space Agency (ESA) Medium Resolution Imaging Spectrometer (MERIS, 2002–2012) onboard Envisat and the two Ocean and Land Color Instruments (OLCI, 2016-present) onboard Sentinel-3A and −3B provide the source of CyAN satellite data records. These ocean color instruments provide global coverage every 2–3 days at ∼300 m resolution. As part of the CyAN effort, mission-long data records for MERIS and OLCI are processed and distributed for the full CONUS and Alaska by the NASA Ocean Biology Processing Group (OBPG; https://oceancolor.gsfc.nasa.gov) at Goddard Space Flight Center.

These satellite data records have been recognized as a valuable tool to reduce public exposure to harmful events by guiding water quality sampling and beach closures in several states such as Utah, Wyoming, Oregon, and New Jersey (Schaeffer et al., 2018; WDEQ, 2019; OHA, 2019; NJDEP, 2020). The utility of the CyAN data set has been further demonstrated in a number of studies that quantify diverse aspects of cyanobacteria blooms across CONUS including the bloom extent, frequency and severity (Clark et al., 2017; Coffer et al., 2020; Mishra et al., 2019; Urquhart et al., 2017). For example, motivated by a human health and drinking water supply concerns, Clark et al. (2017) used CyAN satellite data products to demonstrate the ability of remote sensing to assist with the allocation of limited management resources across diverse lakes and regions. Mishra et al. (2019) established a satellite-based method to quantify seasonal and annual bloom magnitudes for lakes to further support decision making and resource allocation. And, with the goal of quantifying the socioeconomic benefits of remote sensing, Stroming et al. (2020) evaluated the use of CyAN satellite data for monitoring toxic cyanobacteria events in Utah Lake, Utah, a popular recreational waterbody, and estimated that reduced illnesses and improved human health outcomes from such monitoring could result in $55,000 to > $1 million US$ in benefits per cyanobacteria event. Time series of satellite data have been utilized for a variety of inland water analyses to look the evolution of a single bloom event (Wynne et al., 2008), as well as larger area trends on seasonal and annual timescales (Urquhart et al., 2017; Coffer et al., 2020). Using the data set presented here, Coffer et al. (2020) demonstrated that the CONUS bloom season reported in the CyAN satellite data is well-supported in the literature with blooms rising gradually starting late spring and reaching a maximum in late-summer/early-autumn. Mishra et al. (2021) showed the CyAN product had an 84% accuracy for bloom detection based on the ability to match state-reported toxin levels that indicated bloom or no-bloom conditions.

The purpose of this paper is two-fold. First, we describe the production and distribution of the MERIS lakes time-series, which we have made publicly available (https://oceancolor.gsfc.nasa.gov/projects/inlandwaters/). This inland waters data set (ILW) contains 10 years (2002−2012) of observations. ILW will be expanded to include OLCI on both Sentinel-3A (2016-present) and Sentinel-3B (2018-present) as those data become available and are quality controlled (see Appendix B). By design, the distribution of ILW can substantially reduce the processing effort required by end users to work with the MERIS (and eventually OLCI) data for these inland bodies of water and, as such, we offer it as a standardized community resource for future lake and reservoir algorithm development and performance assessment. Second, to highlight the utility of ILW, we offer a case study of *Chl* algorithm development and performance assessment across CONUS, using the Cyanobacteria Index (CI) algorithm; the complete sequence of CI development is detailed in Coffer et al. (2020) and reviewed below. Our purpose in pursuing this case study is not to unequivocally recommend another *Chl* algorithm, as viable alternatives exist, all with their own strengths and weaknesses (Binding et al., 2011, 2013, 2019; Gower et al., 2005; Kutser, 2009; Lesht et al., 2013; Matthews et al., 2012; Matthews and Odermatt, 2015; Moses et al., 2012; Neil et al., 2019, to name only a few). Rather, we pursued this case study to provide a demonstration of how a unified approach, developed using a standardized satellite data set, performs for a diverse set of lakes across the CONUS. A wide diversity of CyAN stakeholders, with varied experience and investment in satellite ocean color, use the daily and weekly imagery distributed by NASA. We believe that demonstrating the development and assessment of *Chl* estimates through this case study may increase stakeholder accessibility, familiarity, use, and comfort with development and refinement of a derived biogeophysical variable. Our ultimate goal, however, remains creating awareness of ILW in the community. This data record is publicly available for use as-is to enable exploration of additional remote sensing algorithm development for U.S. lakes and inland waters.

## 2. Methods

### 2.1. Satellite data processing

Calibrated, geolocated top-of-atmosphere (Level-1B) MERIS data were acquired from the OBPG. The OBPG redistributes this data record through a data sharing agreement between NASA and ESA. The Level-1B data were processed to Level-2 imagery, which have the same projection and resolution as the Level-1 source data, by removing the contribution of spectral Rayleigh scattering from the top-of-atmosphere signal. This Rayleigh-corrected top-of-atmosphere reflectance (*ρ*_s_(λ); unitless) was generated at 413, 443, 490, 510, 560, 620, 665, 681, 709, 754, and 885 nm. ILW includes both *ρ*_s_(λ) and CI_cyano_, discussed in detail below. The spectral remote sensing reflectances *R*_*rs*_(λ) are not provided, as it has been previously demonstrated that the standard OBPG atmospheric correction algorithm underperforms for many inland water bodies (see, *e.g.*, Pahlevan et al., 2017; Warren et al., 2019).

Several processing masks were applied to exclude questionable Level-2 data. An inland waters specific cloud flag was adopted, as the default OBPG ocean processing cloud flag is occasionally triggered by highly reflective waters from blooms or suspended sediments (Wynne et al., 2018). For CONUS, a high resolution (∼60 m) land mask based on the NASA Shuttle Radar Topography Mission Water Body Data Shapefiles (NASA JPL, 2013) was used, with modifications by Urquhart (2018) to correct for embedded inaccuracies in that data set, such as missing lakes and reservoirs in Rhode Island and Massachusetts. As this is a static land mask, a flag for mixed land-water pixels was developed to identify cases where the land mask reported a water pixel, but that pixel did not contain water at the time of satellite observation, which is possible due to the ephemeral spatial extents of inland waters. Finally, flags to indicate potential contamination due to adjacency effects and to identify snow or ice covered water bodies were also applied (Wynne et al., 2018).

CI_cyano_ was calculated from *ρ*_s_(λ) after masking (Eq. (1)). This derivative spectral shape, or line-height, algorithm was selected by the CyAN Project to provide cyanoHAB detection. Through a baseline subtraction that effectively normalizes the absolute signal, line-height algorithms evaluate derivative spectral shape (curvature) in targeted spectral regions – in this case, *Chl* and phycobilin absorption. Line-height algorithms tend to be less sensitive to atmospheric conditions and satellite instrument calibration and data processing artifacts relative to alternative spectral matching and band ratio approaches for *Chl* estimation (Hu et al., 2019). Examples of other common line-height algorithms applied to inland waters include the Maximum Chlorophyll Index (MCI) (Binding et al., 2011, 2013, 2019; Lesht et al., 2013) and the maximum peak height (MPH) (Matthews et al., 2012; Matthews and Odermatt, 2015).

The CyAN implementation of CI proceeds as follows. First, derivative spectral shapes (SS) around 665 and 681 nm are calculated *via*:

(1) $$\text{SS}\left(\text{λ}\right)=\rho_{s}\left(\text{λ}\right)-\rho_{s}\left({\text{λ}}^{-}\right)+\left[\rho_{s}\left({\text{λ}}^{-}\right)-\rho_{s}\left({\text{λ}}^{+}\right)\right]\left(\frac{\lambda -\lambda^{-}}{\lambda^{+}-\lambda^{-}}\right),$$

where the superscripts – and +indicate one sensor waveband less and more, respectively, than the target sensor waveband. The λ^−^, λ, and λ^+^ for MERIS-SS(681) encompasses sensor wavebands 665, 681, and 709 nm, while SS(665) incorporates 620, 665, and 681 nm (Lunetta et al., 2015). These SS are then used in a decision tree to identify cyanoHAB presence. The original implementation defined CI = -SS(681), with a positive CI defined as cyanoHAB presence, following the assumption that cyanobacteria are likely present when *ρ*_s_(681) falls below its baseline value, which results from a combination of insignificant fluorescence and strong chlorophyll *a*bsorption from cyanobacteria at 681 nm (Seppälä et al., 2007 ; Wynne et al., 2008; Binding et al., 2011). Use of SS (681) alone, however, occasionally misidentifies other non-cyanobacteria phytoplankton blooms as cyanoHABs and, furthermore, cannot ubiquitously provide robust estimates of non-cyanobacteria biomass (Matthews et al., 2012; Wynne et al., 2010, 2013). As such, CI was augmented to also consider SS(665) to provide an additional metric for constraining CyAN estimates to cyanobacteria biomass. A spectral shape centered on 665 was used to identify presence of phycocyanin that would separate cyanobacteria from other blooms (Lunetta et al., 2015). This approach was also used by Matthews et al. (2012) for detecting cyanobacteria in African lakes. A positive SS(665) further indicates cyanoHAB presence, following the assumption that phycocyanin absorption depresses *ρ*_s_(620) and alters the curvature around 665 nm. The sign of SS(665) can be used to assign the derived CI value as either CI_cyano_ or CI_noncyano_, with the subscripts indicating the presence of cyanobacteria or not, respectively. ILW only includes CI_cyano_, as the priority of CyAN is cyanoHAB detection, noting that CI and CI_noncyano_ can be easily determined using the provided *ρ*_s_(λ) (Eq. (1)). Wynne et al. (2018) provides additional details on the CyAN suite of products, as well as quality assurance metrics and additional exclusion criteria applied.

As the final ILW processing step, Level-3 composites of *ρ*_s_(λ) and CI_cyano_ for CONUS were generated from the Level-2 imagery using the OBPG’s standard software and processes. This involved generation of Level-3 bin files covering CONUS using an integerized sinusoidal projection (Campbell et al., 1995), followed by production of Level-3 Standard Mapped Images (SMI) using a Plate Carrée projection and nearest neighbor weighting with a 300 m bin size with the bins for the *ρ*_s_(λ) and CI_cyano_ products based on where the maximum CI_cyano_ value is found. Daily imagery was produced for all products for all locations with valid satellite retrievals. The *ρ*_s_(λ) and CI_cyano_ products were further temporally composited as mean values over 7-day (centered on Wednesday), monthly, rolling 28-day, and seasonal ranges. Monthly and seasonal climatologies were also generated. ILW SMIs are provided as complete CONUS and Alaska maps stored as netCDF files, which provide flexibility for a wide range of potential end users. To further ensure accommodation of all potential end users, Appendix C provides details and recipes for reprojecting the SMI imagery into alternate map projections (*e.g.*, Albers conic projection, which is used in operational CyAN processing), as well as for extracting regional spatial subsets and saving the imagery in alternate file formats (*e.g.*, GeoTIFF). All data are publicly available *via* the NASA OceanColorWeb site (https://oceancolor.gsfc.nasa.gov/projects/inlandwaters/). Furthermore, all source code are available through the distribution of SeaDAS.

### 2.2. In situ data

*In situ Chl* measurements based on discrete water samples from the Water Quality Portal (https://www.waterqualitydata.us) were acquired from the USGS CyAN Field Integrated Exploratory Lakes Database (Eslick et al., 2019). The *in situ* data were for CONUS only. The initial search for *Chl* data from 2002 to 2012 resulted in 547,783 measurements. After filtering these data using the criteria described below, the sample size reduced by 67%, leaving a final count of 148,018 measurements for use in this study. Data filtering used the following steps: (1) samples collected at >0.5 m depth were discarded, as were those lacking a reported depth value, to ensure that only near-surface samples were considered; (2) negative *Chl* values and extremely high values (> 2000 μg L^−1^) were discarded, as they are extreme outliers for this data set, as well as outside the range for meaningful satellite detection; (3) samples with different reported start and end dates were removed, as it was impossible to ascertain the actual collection time; (4) only sample types labeled “Sample-Routine,” “Field Msr/Obs,” or “Sample,” were retained, as other sample type designations indicate measurements for laboratory quality control that are not appropriate for validation; and (5) replicate samples with identical dates, times, locations and depths were removed. The linear distance from an *in situ* sampling station to the nearest shore was estimated from each sample’s coordinates using a revised version of the National Hydrography Dataset Plus lakes shapefile (NHDPlus v2.0 polygons; U.S. Geological Survey, 2012). Only samples collected >300 m from shore were retained to minimize potential inclusion of land-water pixels or those contaminated by adjacency effects or bottom reflectance in optically shallow water.

### 2.3. Chl algorithm case study

It has long been established that the NIR region of the spectrum has a meaningful relationship with *Chl* concentration (Gitelson, 1992). Tomlinson et al. (2016) demonstrated the use of CI to estimate *Chl* in high chlorophyll lakes in Florida. Here, we extend this effort to explore the use of CI_cyano_ to estimate *Chl* across the CONUS. The Tomlinson et al. (2016) formulation is:

(2) $$Chl_{T16}=4050\left(\pm 271\right)\times CI+20\left(\pm 3\right)$$

Their training data set focused on cyanobacteria dominated lakes and included remote sensing reflectances from above water radiometers for the calculation of CI and *in situ Chl* measurements ranging from eutrophic to hypereutrophic conditions (16 to 115 μg L^−1^). Tomlinson et al. (2016) reported a bias of 3 μg L^− 1^ and a root mean square error (RMSE) of 15 μg L^−1^ for *Chl*_T16_ with a relative RMSE of 27%. They calculated CI (not CI_cyano_), but as these Florida lakes were cyanobacteria-dominated their CI is expected to be comparable to the CI_cyano_ used throughout this paper.

Our *in situ* data set spans oligotrophic to hyper-eutrophic conditions (0.003 to 750 μg L^−1^), which presents an opportunity to re-tune a CI to *Chl* algorithm to a wider range of conditions more representative of CONUS lakes. Our re-tuning considered MERIS-derived CI_cyano_ and *in situ Chl*. This required accumulating satellite-to-*in situ* match-ups. We acquired Level-3 MERIS-to-*in situ* match-ups from the OBPG following the methods of Scott and Werdell (2019). Briefly, this involved retrieving Level-3 daily SMIs from ILW, where a valid satellite pixel matched an *in situ* target on the same day the *in situ* sample was collected. This resulted in 1738 MERIS-to-*in situ* match-ups available for final analyses (Fig. 3).

A bootstrapping approach was used to relate the paired MERIS CI_cyano_ with *in situ Chl* following Eq. (2). Bootstrapping creates a series of data sets *via* random sampling from and replacement to a larger, original data set (Efron, 1979). Bootstrapping assumes the full data set represents the population of interest and follows an iterative sampling-with-replacement strategy, where data selected for a sub-sampled data set are returned to the original full data set for potential reuse in a subsequent sub-sampled data set. A variety of bootstrapping approaches exist, and selection of method requires consideration of data set characteristics such as sample size and distribution (Efron and Tibshirani, 1997; Davison and Hinkley, 1997; Fox, 2002; Bi et al., 2021). For the bootstrapping used in this analysis, the 1738 satellite-to-*in situ* match-ups were split into two data sets – a training data set consisting of 80% of the match-ups and an evaluation data set consisting of the remaining 20% (Fig. 3). The bootstrapping sample-with-replacement method was applied to the training data set for 1500 iterations (Python with Scikitlearn, a machine learning library). In each iteration, a regression analysis was executed to estimate the algorithm coefficients (Eq. (2)). Analysis of the full suite of runs allowed derivation of average coefficients and their 95% confidence intervals. The performance of the newly tuned bootstrapped *chl* algorithm, *Chl*_BS_, was assessed with the evaluation data set (the isolated 20%). In the context of algorithm development, this process of resampling and exposing the algorithm to fits from multiple data sets offers advantages relative to a single linear regression fit on the full data set. It not only reduces the risk of overfitting to one particular data set, but also allows production of confidence intervals around the coefficients.

### 2.4. Performance assessment

Ocean color algorithms are often validated using least squares regressions and analysis of their errors (*e.g.*, Werdell and Bailey, 2005). Our satellite-to-*in situ* data set is not normally distributed (Fig. 4). Therefore, mean square error statistics were not reported, as they are best suited for Gaussian distributions. Instead, we focus on mean bias and mean absolute error (MAE)to summarize algorithm performance (Seegers et al., 2018):

(3) Biaslog=10^(Σj=1nlog10(Mi)−log10(Oi)n)

(4) MAElog=10^(Σi=1n|log10(Mi)−log10(Oi)|n)

where *M*, *O*, and *n* represent the modeled satellite value, the *in situ* observation, and the sample size, respectively. Log-transformed metrics were used (Eqs. (3) and (4)), because error for *Chl* is heteroskedastic and the data ranges four orders of magnitude, which is common in the validation field (Seegers et al., 2018). Also, the log approach helps minimize analytical biases and the influence of outliers (Tofallis, 2015). The metrics, as shown, are based on geometric mean, converted from log units and are dimensionless. Their interpretation is roughly multiplicative, meaning that a bias_log_ of 1.3 indicates that the model is 1.3× (30%) greater on average than the observed variable, while a bias less than unity indicates a negative bias. MAE_log_ always exceeds unity, such that a MAE of 1.2 indicates relative measurement error of 20% in either direction. While the term “error” is used here as it is in the vast majority of validation studies, it is acknowledged that all are actually referring to “misfit”, because the reference data also have observation errors and therefore the actual error is not quantifiable (Lynch, 2009).

In the context of water quality monitoring, many stakeholders are additionally interested in broadly categorizing performance to simply determine “is there a problem?” and, if yes, “how bad is it?” (IOCS, 2015). Thus, performance was additionally assessed by quantifying the frequency for which the satellite retrieval properly identified the trophic category into which each corresponding *in situ* data point belonged. To do so, the *in situ* value was used to divide the match-up data set into four trophic categories based on criteria from the National Lakes Assessment: oligotrophic/mesotrophic (0–7 μg L^−1^), eutrophic (7–30 μg L^−1^), and two hypereutrophic conditions (>30 μg L^−1^) (U.S. Environmental Protection Agency, 2009). Because of its large range (30–650 μg L^−1^), the hypereutrophic category was further divided into “low hypereutropic” (30–90 μg L^−1^) and “high hypereutrophic (>90 μg L^−1^). High hypereutrophic conditions (>90 μg L^−1^) fall in the top 25% of data points in terms of *Chl* concentration in both the training and evalutation data sets. A confusion matrix was generated to report percentages of correct trophic level characterization by *Chl*_BS_. Generally, a confusion matrix is a visual display of an algorithm’s ability to properly predict categories.

## 3. Results

### 3.1. The composition of ILW

ILW provides data across CONUS for over 2300 resolvable lakes with sizes greater than three 300 m pixels (Urquhart and Schaeffer, 2019; Coffer et al., 2020) and 15,450 waterbodies with sizes of at least one 300 m pixel (Clark et al., 2017) and 5874 lakes of at least one pixel size in Alaska (Fig. 1). ILW currently consists of L2 files, L3-binned files, and L3 SMIs spanning 28 April 2002 to 9 April 2012, representing the mission life of MERIS. The total file volume for this first version of ILW is greater than 20 TB with 16 TB of L2 *ρ*_s_(λ) data. The remaining 4 TB are L3-mapped and L3-binned files which provide 3600 daily files, plus 527 files for both weekly and rolling 28-day files, 120 monthly files and 48 seasonal files. Occassionally, ILW will be reprocessed, so the exact file numbers will change and version numbers will be given to updated versions. Additionally, OLCI will be added to the ILW data set, which will greatly expand the time series.

**Fig. 2.**
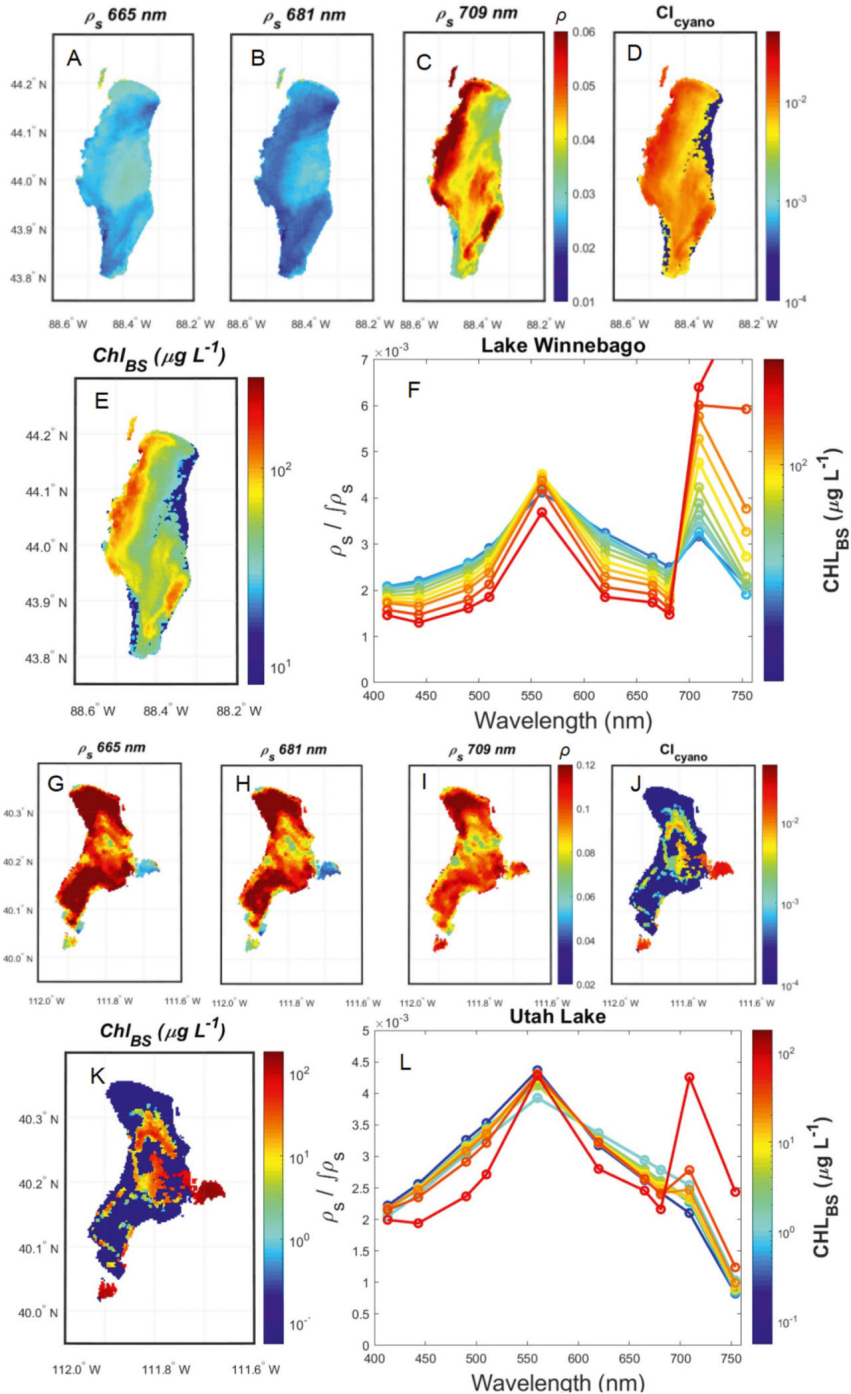
A comparison of 10 September 2011 satellite data from two different lakes in different U.S. regions; Lake Winnebago, Wisconsin (top; A-F) and Utah Lake, Utah (bottom; G-L) showing mapped satellite images of *ρ*_s_ (665, 681, 709 nm; A-C, G-I), CI_cyano_ (D,J) and *Chl*_BS_ (E,K) and *ρ*_s_(λ) spectra (F,L). The maps of each lake’s CI values and corresponding *Chl*_BS_ demonstrate different water types present (D,E,J,K). For each lake the median MERIS *ρ*_s_(λ) spectra are shown for pixels in discrete *Chl* ranges with colors representing diverse water types based on discretized ranges of /*Chl* values (F,L). To focus on the variations in spectral shape, and not spectral amplitude, each *ρ*_s_ spectrum was normalized by its integrated value [*ρ*_s_(λ) / *∫ρ*_s_] over the range of 400–754 nm. These spectra are included as part of the ILW suite. The circles along the lines mark the center wavelength of measured satellite bands (F,L).

Lakes can have wide-ranging MERIS *ρ*_s_(λ) spectra. Lake Winnebago, Wisconsin and Utah Lake, Utah are from ecologically distinct regions of the USA, the upper Great Lakes Region and the Southwest, respectively. These lakes were selected for closer illustration of how the varied line heights relate to a dynamic range of CI_cyano_ retrievals as well as to provide examples of MERIS imagery and *Chl*_BS_ retrievals (Fig. 2). Level-3 Standard Mapped Images (SMI) from ILW were obtained for September 10, 2011, showcasing cloud-free scenes for Lake Winnebago, WI and Utah Lake, UT. Appendix B demonstrates the same products displayed for OLCI on Sentinel-3A and Sentinel-3B. The selected lakes exhibit divergent *ρ*_s_(λ) spectra, yet the line height algorithms allow for meaningful interpretation of the data across the waterbodies as demonstrated in mapped imagery for both the CI and *Chl*_BS_ algorithms (Fig. 2). The variations in spectral shape were highlighted by normalizing each *ρ*_s_ spectrum by its integrated value [*ρ*_s_(λ) / *∫ρ*_s_]. The integration was calculated over the 400–754 nm range using the trapezoidal rule.

### 3.2. The Chl algorithm development data set

**Fig. 3.**
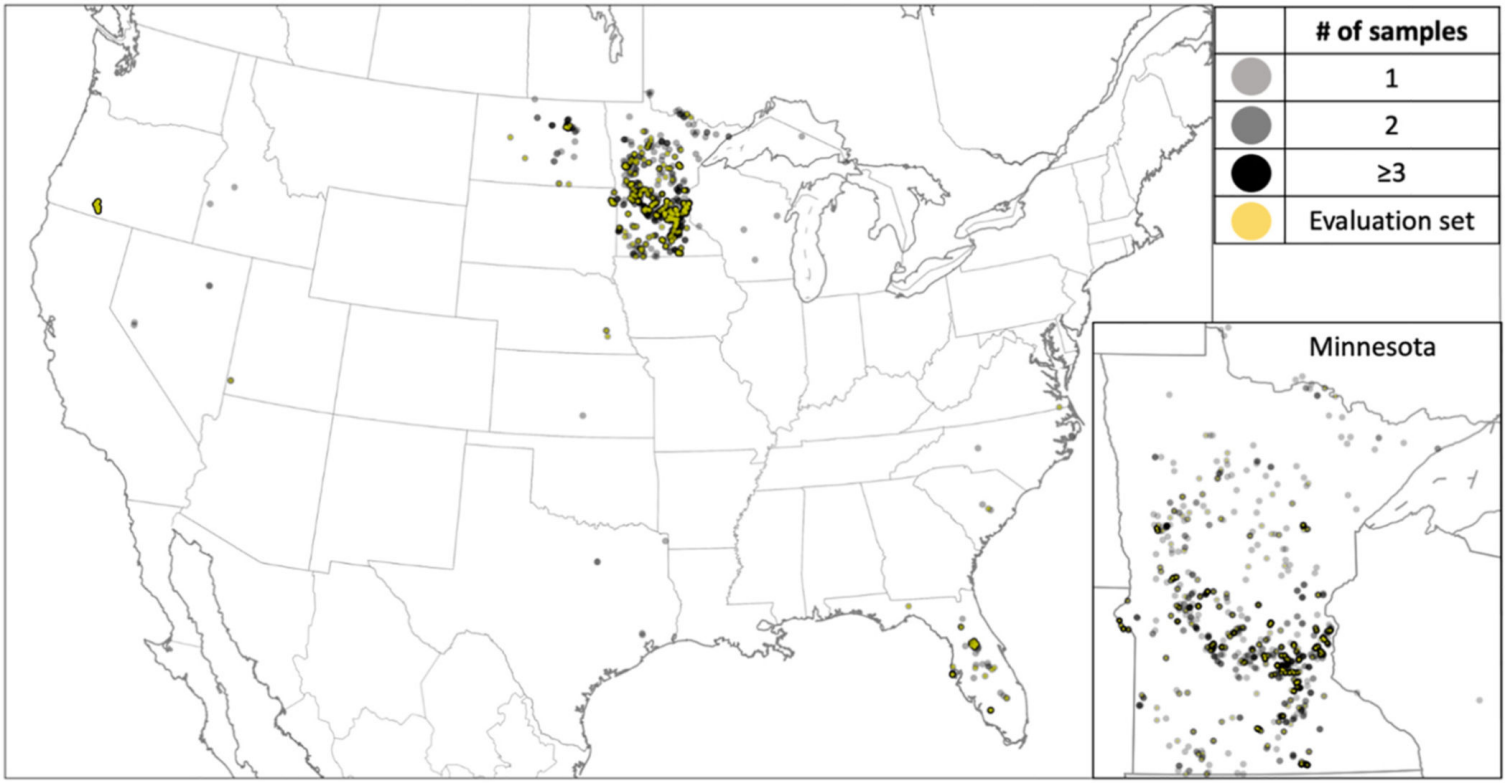
CONUS match-up locations. The light grey circles represent a single sample and black represents ≥3 samples at a location. 15 states have at least one sampling location. The inset map shows match-up locations in Minnesota, which is the location for 72% of the match-up results. The yellow indicates the location of a sample that was part of the 20% of samples used for evaluating the *Chl*_*BS*_ algorithm. (For interpretation of the references to color in this figure legend, the reader is referred to the web version of this article.)

**Fig. 4.**
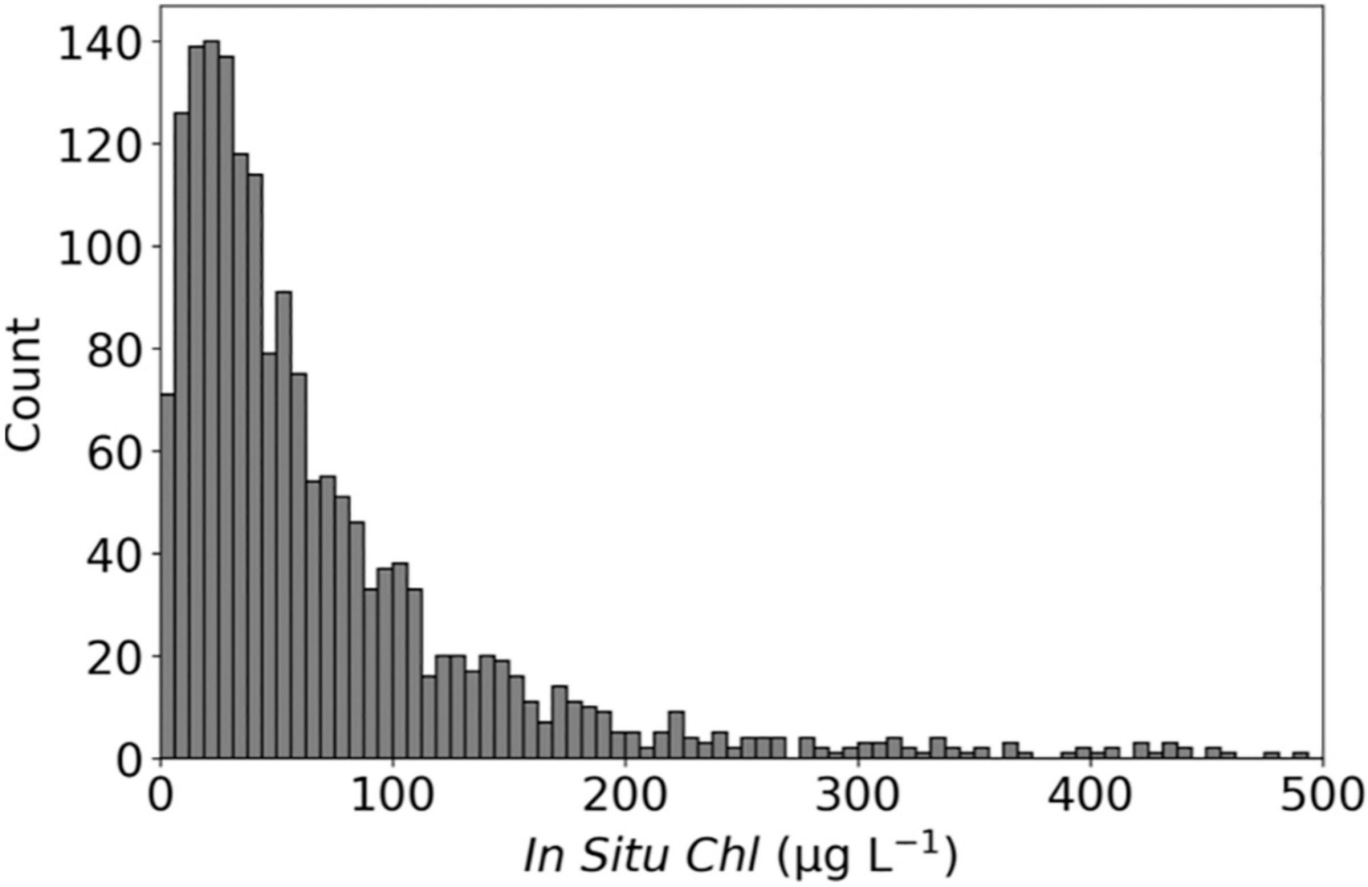
Data distribution of the 1738 *in situ* values from the match-up results.

**Fig. 5.**
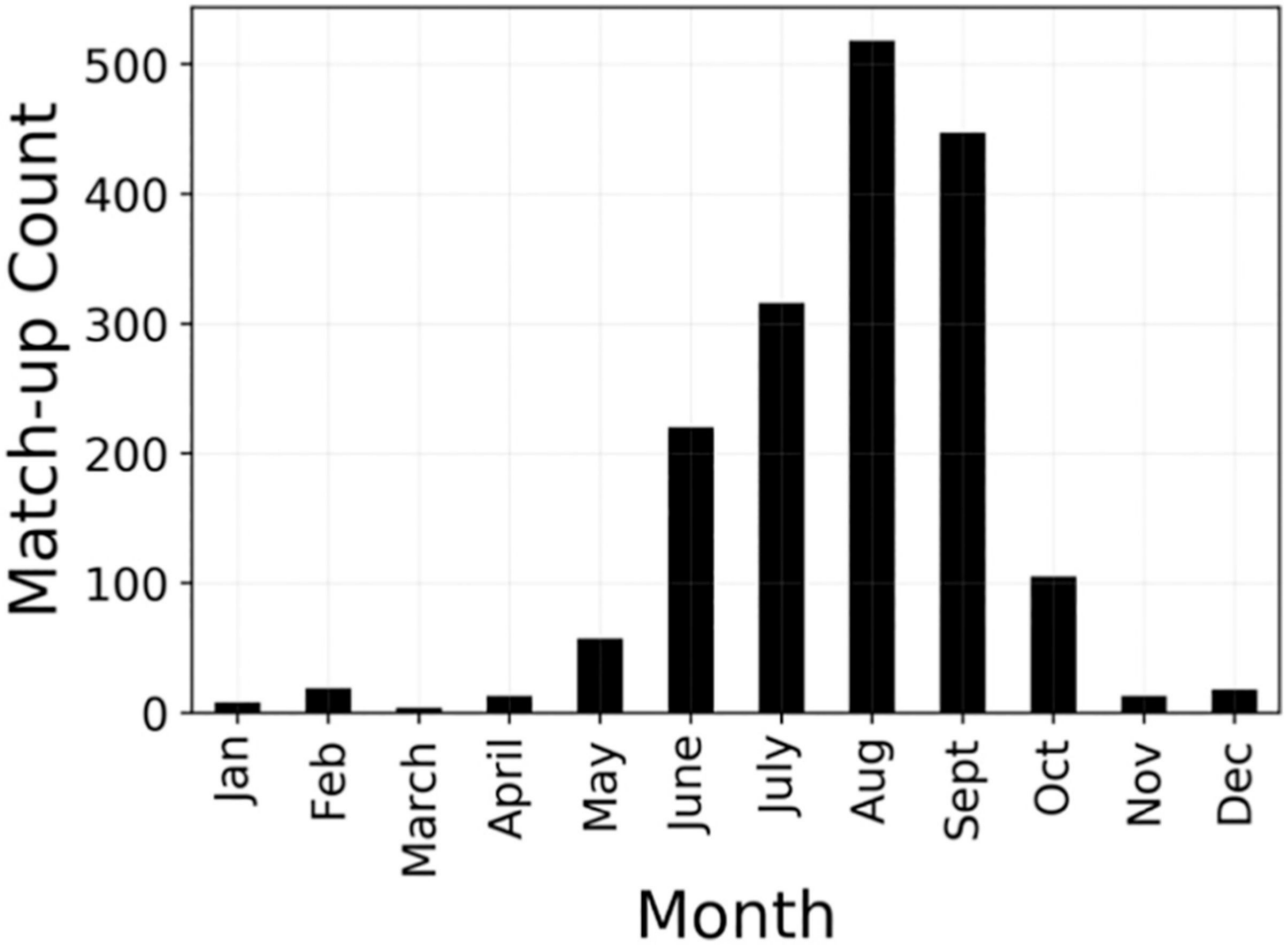
The monthly distribution of the 1738 *in situ* and satellite match-up results. 95% of all match-ups occurred between May and October.

The final filtered ILW CI_cyano_-to-*in situ Chl* match-up data set (https://oceancolor.gsfc.nasa.gov/fileshare/jeremy_werdell/CyAN_ChlBS/) included 1738 match-ups from 15 states across CONUS (Minnesota: 1263; Oregon: 291; Florida: 98; North Dakota: 51; Texas: 5; Nevada: 4; Nebraska, North Carolina, South Carolina, Wisconsin: 3; Idaho, Michigan, Utah:2; Kansas, Virginia: 1) (Fig. 3). The majority of the match-ups occurred in Minnesota (72%) and Oregon (17%), while the remaining 13 states made up 10% of the data (Fig. 3). Ideally, the match-ups would be more evenly spread across the country, however, Minnesota has more satellite resolvable lakes than any other state, with 17.5% of all CONUS resolvable lakes located in that state (Schaeffer et al., 2018). Minnesota also provided one of the larger volumes of *in situ* data, making satellite matches more probable. Fortunately, Minnesota has diverse lakes with *in situ Chl* concentrations included in this analysis ranging from 0.51 to 650 μg L^−1^. Additionally, the region includes farmland, urban systems, and forested watersheds providing varied aquatic systems for algorithm testing. The full match-up data set included *in situ* measurements across water types from oligotrophic to hypereutrophic, with *Chl* ranging from 0.5 to 832 μg L^−1^, a mean concentration of 71.2 μg L^−1^, and a median concentration of 45 μg L^−1^. The vast majority (79%) of these match-ups included *in situ* values with *Chl* < 100 μg L^−1^ (Fig. 4). The summer and autumn seasons have the highest frequency of match-ups with 96% occurring between May and October and 56% falling into the two months of August and September (Fig. 5).

### 3.3. Performance of Chl_T16_

**Fig. 6.**
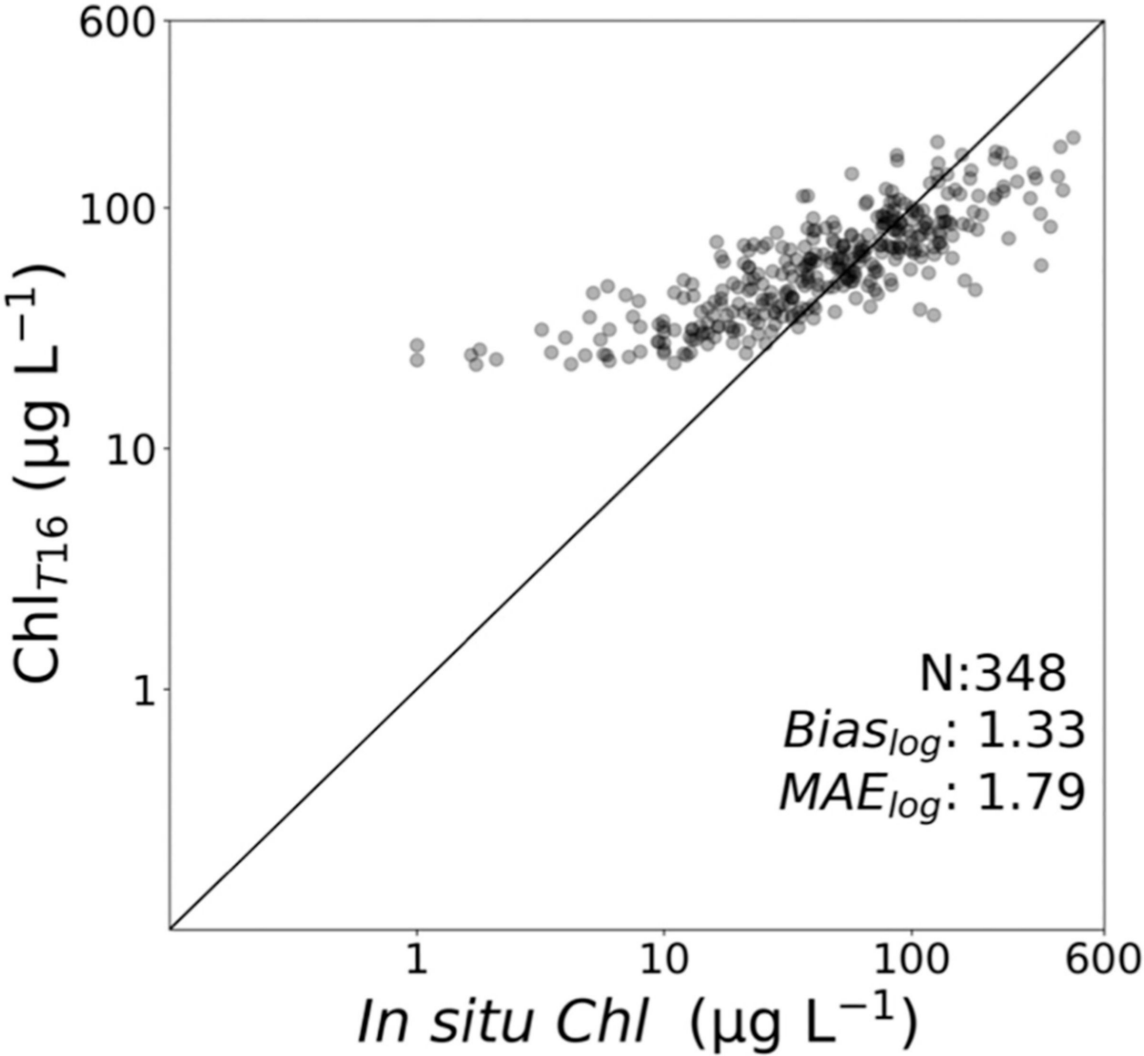
Comparison of *in situ Chl versus Chl*_T16_ retrievals (Tomlinson et al., 2016). The shading indicates the data density with dark colors being more data points compared to the lightly shaded markers.

When applied to the evaluation satellite-to-*in situ* data set assembled in this study (20% of all available data), *Chl*_T16_ reported a positive bias_log_ of 1.33 (33%) and MAE_log_ of 1.8 (80%), (Fig. 6, Table 1). Only the evaluation data set was considered here, for consistency with *Chl*_BS_ analysis. It is clear from Fig. 6 that *Chl*_T16_, which was developed primarily for hypereutrophic lakes in Florida, provides meaningful *Chl* retrievals when the analysis focuses only on high chlorophyll conditions (>20 μg L^−1^), with the bias_log_ dropping to 1.01 (1%) and MAE_log_ dropping to 1.48 (48%, Table 1) for this range. For the eutrophic category, *Chl*_T16_ reported a positive bias_log_ and MAE_log_ of 2.23 (123%) and 2.2 (120%), respectively. Performances in both hypereutrophic categories exceeded that of the eutrophic category. The low hypereutrophic category reported a reduction of bias_log_ to 1.16 (16%) and MAE_log_ to 1.3 (30%). A tendency to underestimate *Chl* in the high hypereutrophic category yielded a negative bias_log_ of 0.60 (− 40%) and MAE_log_ of 1.7 (70%).

The large MAE_log_ and bias_log_ in the oligotrophic range is unsurprising as Tomlinson et al. (2016) reported a minimum detection level of ∼20 μg L^−1^ for *Chl*_T16_. Their intercept reflects the inherent eutrophic nature of these lakes, as well as a potential background concentration of *Chl* from other (non-cyano) phytoplankton that may be as much as 20 μg L^−1^. Their approach, therefore, does not accurately assess low concentrations and should not be applied under these conditions, which explains why eliminating the lowest *Chl* concentration locations led to improved performance.

### 3.4. Development and performance of Chl_BS_

**Fig. 7.**
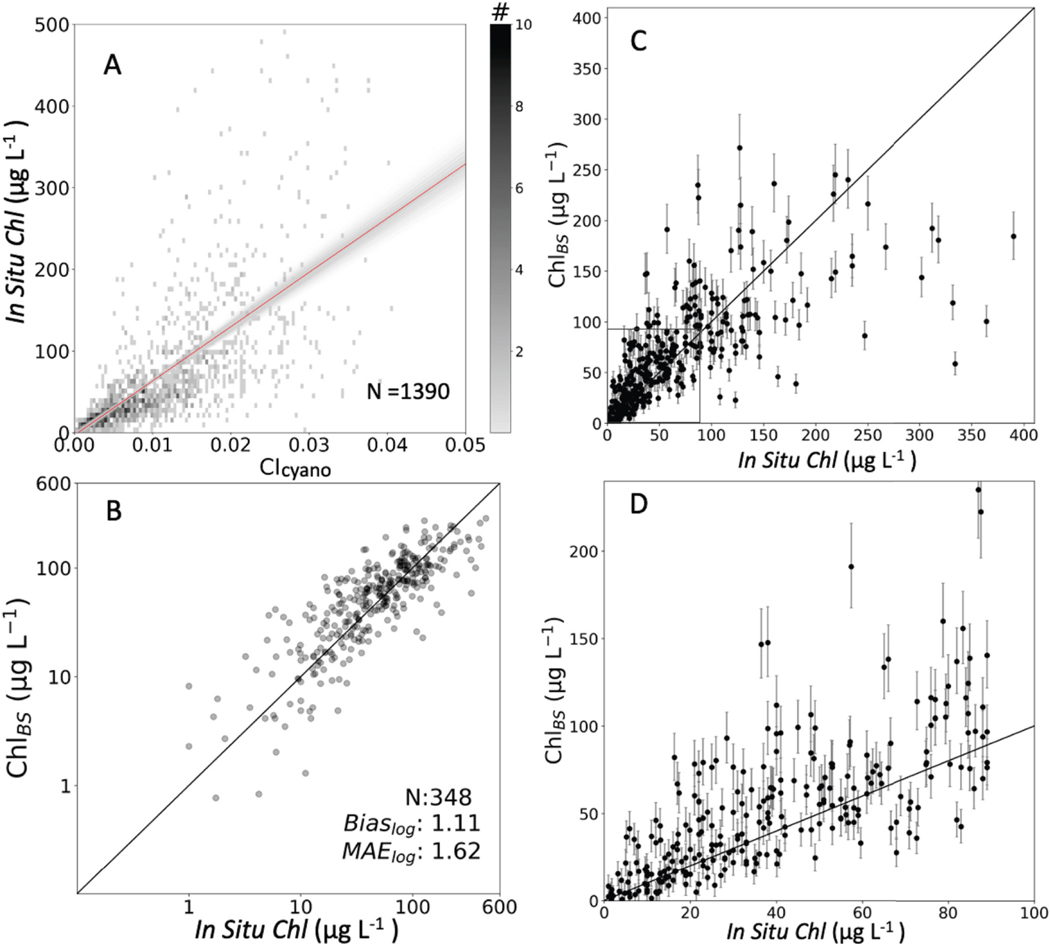
(A) Bootstrap results for 1500 iterations of the CI_cyano_ training data set. The red line is the mean fit and the grey shows the spread of fits. The color shading indicates data density with light grey representing fewer data points compared to black indicating high data density. (B) Predicted *Chl*_BS_ *versus* evaluation data set *in situ Chl* concentrations with log-transformed axes. The grey to black shading is an indication of data density from low to high. (C) Results from panel B in normal space in the range 0–650 μg L^−1^). The bars indicate the potential uncertainty range for each predicted data point based on the *Chl*_BS_ coefficients’ 95% confidence intervals. (D) Exploded view of the data from the box in the lower left of panel C allowing easier viewing of the high density, low *Chl* (0–90 μg L^−1^) data. (For interpretation of the references to color in this figure legend, the reader is referred to the web version of this article.)

The bootstrapping training data subsets, consisting of 1390 data points (80% of the full data set), were run through 1500 bootstrapping iterations (Fig. 7A), resulting in the following relationship:

(6) $$Chl_{BS}=6620\left(\pm 646\right)\times CI_{cyano}-3.1\left(\pm 5.2\right)$$

While the intercept is negative, we note that its standard deviation is larger than its value, indicating that the intercept is not meaningfully different than zero. That said, we would advise caution when considering *Chl*_BS_ retrievals that approach null to avoid biases. While admittedly imperfect to do so, we considered removal of all negative *Chl*_BS_ retrievals in our subsequent analyses. For these data, however, no negative values were reported and, therefore, no match-ups were removed from consideration.

The performance of *Chl*_BS_ was assessed using the evaluation data set, consisting of 348 data points (20% of the full data set). *Chl*_BS_ reported a slight positive bias_log_ of 1.11 (11%) and MAE_log_ of 1.6 (60%), both of which improve upon the performance of *Chl*_T16_ (Table 1; Fig. 7B)_._ The improved performance of *Chl*_BS_ relative to *Chl*_T16_ was anticipated, as it was trained from a larger data set over a wide range of data values. The removal of data points < 20 μg L^−1^ improved the bias_log_ with a slight reduction to 1.04 (4%) and the MAE_log_ improved to 1.52 (52%) (Table 1). The poorest performance was seen in the oligotrophic/mesotrophic range with a bias_log_ of 1.79 (79%) and a MAE_log_ of 2.8 (180%). Performance in the eutrophic range gave a bias_log_ of 1.27 (27%) and a MAE_log_ of 1.8 (80%). *Chl*_BS_ yielded the lowest MAE_log_ of 1.4 (40%) in the low hypereutrophic range with a positive bias of 1.19 (19%). The tendency to underestimate in the high hypereutrophic range resulted in a negative bias of 0.73 (− 27%) and a MAE_log_ of 1.5 (50%). When considering the full range of data *Chl*_BS_ was an improvement over *Chl*_T16_ with a reduction in both bias_log_ and MAE_log_. However, the improvements were not uniformly seen across all the concentrations. For example, in the 20–700 μg L^−1^ range and the low hypereutrophic range *Chl*_BS_ and *Chl*_T16_ performed similarly.

The bootstrapping approach resulted in a spread of potential curve fits (Fig. 7A) allowing for the calculation of coefficient confidence intervals. Following, the 95% confidence intervals for the *Chl*_BS_ coefficients were determined (Eq. (6)). The evaluation data set was plotted using lines to indicate the spread of predicted *Chl* values that would result from using the full range of coefficients defined by the 95% confidence intervals (Fig. 7C,D). Clearly, the spread grows with increasing predicted *Chl* concentrations reflecting the increasing uncertainty. Relatively small confidence intervals, compared to the coefficient, indicate a strong algorithm fit, while larger confidence intervals imply a weaker relationship. As Fig. 7A shows there is more spread in the curve fit at the high end where there are far fewer data points. If there was a desire for a tighter fit, more data at the high end could be useful.

**Fig. 8.**
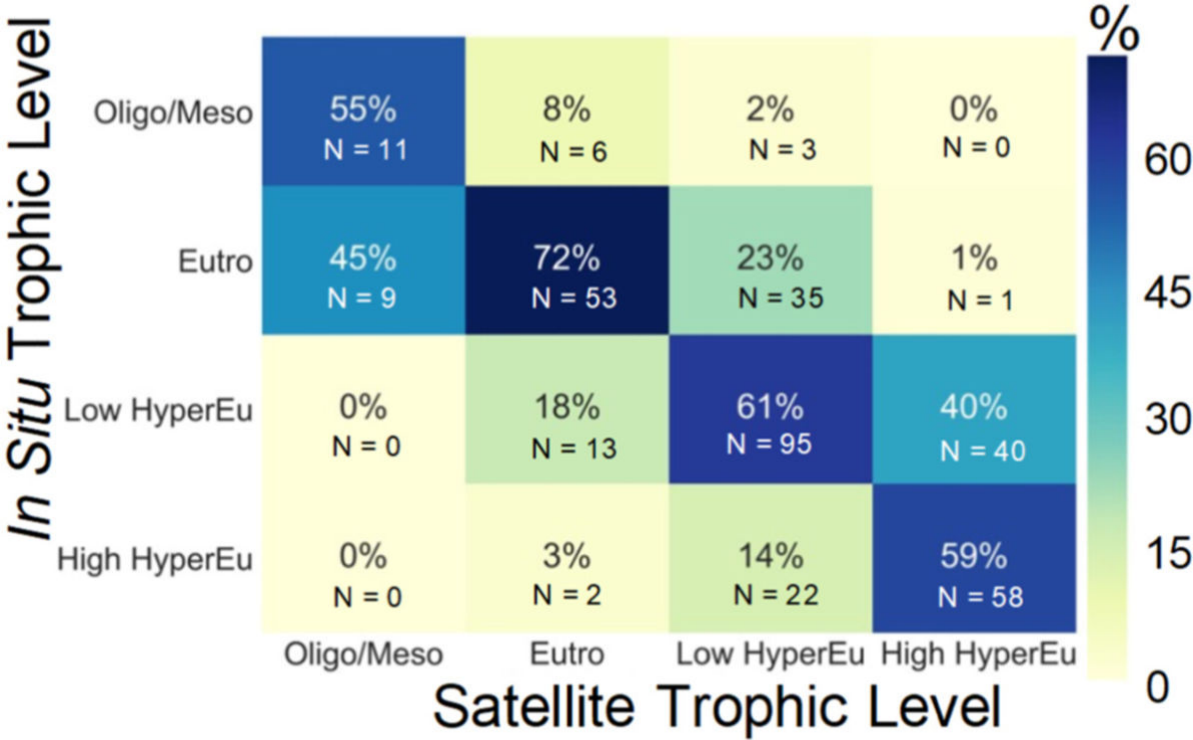
Confusion matrix percentages to illustrate the frequency of which the algorithm, *Chl*_BS_, predicts the trophic status of the *in situ* sample placed into 4 broad categories of oligotrophic/mesotrophic (0–7 μg L^−1^), eutrophic (7–30 μg L^−1^), low hypereutrophic (30–90 μg L^−1^), and high hypereutrophic (> 90 μg L^−1^).

A summary of the ability of *Chl*_BS_ to assess four broad categories of trophic status, specifically, oligotrophic/mesotrophic, eutrophic, low hypereutrophic, and high hypereutrophic, is also presented in the form of a confusion matrix (Fig. 8). The algorithm minimum detection limit is an improvement over *Chl*_T16_ minimum detection level, but nonetheless *Chl*_BS_ still results in an overestimation of *Chl* in low concentrations. In the 0–7 μg L^−1^ range *Chl*_BS_ properly assigned the category in 55% of cases and overestimated 45% of the time. The *Chl*_BS_ best performance is in the eutrophic range correctly predicting the trophic state in 72% of cases, overestimating 8% of cases oligotrophic/mesotrophic cases that were miscategorized as eutrophic, and underestimating 21% of hypereutrophic cases categorized as eutrophic (Fig. 8). The breakdown for the low hypereutrophic (30–90 μg L^−1^) range shows that low hypereutrophic cases were identified correctly 61% of the time, while overestimation resulted in lower *Chl* cases being miscategorized into the low hypereutrophic category 25% of the time of which 23% were eutrophic cases and 2% were in the oligotrophic/mesotrophic range. Underestimation led to 14% of cases being miscategorized as low hypereutrophic rather than high hypereutrophic. The *Chl*_BS_ in high hypereutrophic range correctly predicted 59% of samples, while 40% were overestimated and categorized as high hypereutrophic rather than low hypereutrophic (Fig. 8). When all hypereutrophic waters, that is, all cases >30 μg L^−1^, were analyzed together, then 85% (215 out of 254) of the *Chl*_BS_ trophic category matched the *in situ* measurement. Overall, *Chl*_BS_ showed promise at trophic status identification.

## 4. Discussion

Our primary motivation for this work was creating awareness that the CyAN Project developed and is distributing the mission-long MERIS time-series of CONUS and Alaska lakes data, inclusive of core radiometric products and the CI_cyano_ optical data product. Similar time-series from OLCI on both Sentinel-3A and −3B will be developed and distributed when data are properly quality controlled, possibly by the time of publication of this work (https://oceancolor.gsfc.nasa.gov/projects/inlandwaters/). Given growing interest in satellite-based water quality monitoring, straightforward access to such data has become increasingly critical to support the emerging cohorts of new end-users and stakeholders. The distribution of this standardized and consolidated data set offers two substantial contributions to the water-quality monitoring communities. First, MERIS radiometric data for CONUS and Alaska have been processed to Level-2, projected onto a Plate Carrée Level-3300-m grid, and included in this distribution. To our knowledge, such a data set has not been previously compiled in such a manner. This allows algorithm developers motivated to pursue alternative methods to relate principle ocean color remote-sensing radiometric variables to any inland water biogeophysical data product of interest. We envision this radiometric data set could be used to evaluate existing inland remote sensing approaches, to enable new performance assessments as additional *in situ* data become available, and to support regional tunings and reparameterizations. Fig. 2 shows divergent *ρ*_s_ spectra across water types, demonstrating that radiometric distinction is possible and gives reason for optimism in the utility of this data set for inland waters. Although Lake Winnebago and Utah Lake show varied spectra, the utility of the satellite data to be able to map meaningful biophysical variables of concern such as chlorophyll concentrations and potentially dangerous harmful algal blooms was demonstrated. The spatial distribution of phytoplankton could provide meaningful information for water managers concerned about human or ecosystem health. This demonstrates the power of satellite data for inland water use and creates awareness of its untapped potential to further develop approaches and algorithms for regional to global applications. Previously, processed satellite data were not easily available for inland waters and perhaps the most useful aspect of this contribution is that this data set removes much of the burden of satellite data processing for end-users and stakeholders.

Our secondary motivation was to explore the viability of using a single algorithm to estimate a biogeophysical variable across the spatially and temporally diverse CONUS data record. We explored using the readily available CyAN optical metrics of cyanobacteria presence (CI_cyano_) as a rudimentary estimate of chlorophyll biomass (*Chl*_BS_). This serves end-users with interest in the CyAN Project’s core biogeophysical deliverables and offers a performance assessment of their quality and utility across a spatially and temporally diverse data set. Perhaps more importantly, it also provides less familiar end-users a roadmap for using ILW in algorithm development, while demonstrating the challenge of universally relating an optical property to a biological or biogeochemical one. Acknowledging, of course, the need for many end-users and stakeholders to operate and communicate using biogeophysical variables in lieu of optical variables. A line-height calculation stemming directly from measurements of reflectance, CI_cyano_ is expected to be, in principle, universally applicable (assuming adequate consideration of Rayleigh calculations, mixed land-water pixels, clouds, snow/ice, and adjacency effects). Its relationship to any biogeophysical condition, however, requires conscientious consideration. Given here was a demonstration of methods and pitfalls to avoid that should provide a useful roadmap and foundation with which to support an emerging cohort of end-users.

Regionally, *Chl* derived from CI has been shown to be meaningful (Tomlinson et al., 2016). Arising from the high *Chl* conditions from which it was developed, *Chl*_T16_ has an intercept of 20 μg L^−1^. *Chl*_T16_ is based on local above water radiometry from Florida eutrophic and hypereutrophic lakes with data collected in one summer, thereby covering a narrow set of conditions. The intercept is likely an offset from the background chlorophyll not associated with cyanobacteria, which is unlikely in most lakes, although relevant to certain hypereutrophic Florida lakes. This offset has been adjusted for other high *Chl* regional applications including to 10 μg L^−1^ in Lake Erie (Rowe et al., 2016) demonstrating the utility of regional tuning. It is encouraging that the *Chl*_BS_ slope coefficient of 6620 is the range of the more regional specific tuned *Chl*_T16_ slope of 4050 giving confidence in the CI_cyano_ to *Chl* relationship. Other studies have also successfully demonstrated the relationships of line-heights to biogeophysical variables, primarily on a regional basis (Binding et al., 2011, 2019; Lesht et al., 2013; Matthews et al., 2012; Matthews and Odermatt, 2015). We explored the robustness of a universal CONUS application, which served to demonstrate how well a single algorithm can perform across spatially and optically diverse and complex waters. While both *Chl*_T16_ and *Chl*_BS_ provide valuable information, their performance assessments show room for improvement and reiterate the need for additional development of inland water algorithms to expand the accuracy and applications of satellite remote sensing in these systems. Distribution of the ILW data set can support such efforts. For many end-user applications, *Chl*_BS_ performance may be perfectly adequate for early warning detection or trend detection, not unlike the previous studies that used CI_cyano_ cyanobacteria estimates (Clark et al., 2017; Urquhart et al., 2017; Mishra et al., 2019; Coffer et al., 2020). For other applications, regional reparameterizations or alternative approaches may be most prudent. Neil et al. (2019) reviewed 19 *Chl* algorithm and 48 approaches to applying the algorithms for 13 different water types and proposed that an adaptive framework leads to overall improvement of estimates. However, this dynamic method of algorithm selection, while potentially more precise, may not be ideal for users who only require a simplified approach for broad water quality monitoring and response. Again, our case study serves to provide a recipe for simple algorithm development for a stakeholders who wish to generate an alternative CI_cyano_-to-biogeophysical variable relationship.

Generally speaking *Chl*_BS_ showed utility in estimating chlorophyll concentrations across CONUS lakes (Table 1). Our results support the Matthews et al. (2012) finding that such algorithms are suitable for trophic status assessment and appropriate for providing some warning signs for harmful algal bloom (HAB) events. However, there remains uncertainty about the precise retrieved quantity of *Chl* and, therefore, analyses that require constraining small changes may not yet be able to consider satellite retrievals. The *Chl*_BS_ algorithm tended to perform best in the >7 μg L^−1^ range, while underperforming at the lowest chlorophyll concentrations (oligotrophic-mesotrophic, Table 1). The reduction in performance in the low end could be caused by a number of variables. First, the ρ_s_(λ) signal in the NIR must overcome the absorption of pure water in that range. The CI_cyano_ 709 nm peak and the associated negative 681 nm curvature result from cyanobacteria scattering, chl-a absorption and limited cyanobacteria fluorescence compared to other phytoplankton in the 665 to 709 nm range and at low cyanobacteria concentrations the signal may not be strong enough for 681 nm spectral shape to develop (Gower et al., 1999; Wynne et al., 2008). Furthermore, at very low signal levels, satellite instrument performance (*e.g.*, signal-to-noise characteristics) in the NIR could confound meaningful retrievals. Ultimately, the reduced performance in the low concentration range limits confidence in assessments of modest changes in water quality in low *Chl* waters. Such limits in the low *Chl* range are not unique to *Chl*_BS_ and have been previously reported as common for chlorophyll *a*lgorithms using red and near-infrared radiometric measurements (*e.g.* Binding et al., 2013; Gilerson et al., 2010; Moses et al., 2012; Palmer et al., 2015). Neil et al. (2019) also acknowledged the limitations of red/near-infrared *Chl* algorithms at low concentrations and suggested a switching approach using a blue-green band ratio algorithm (*e.g.*, O’Reilly and Werdell, 2019) in oligotrophic systems and a red/NIR method in systems with concentrations spanning 3–155 μg L^−1^. Gilerson et al. (2010) observed that *Chl* algorithms using the red/NIR portion of the spectrum outperform blue-green band ratio algorithms at concentrations greater than 5 μg L^−1^. Binding et al. (2019) also found that the MCI and CI worked better than band-ratio approaches for *Chl* > 10 μg L^−1^. Additionally, *Chl*_BS_ tends to underestimate the highest concentrations (>90 μg L^−1^) (Table 1). This is also consistent with Neil et al. (2019), which suggested alternative approaches at high concentrations >155 μg L^−1^. Ultimately, we propose that end-users interested in the trophic state of a lake can use *Chl*_BS_ to provide a functional state of the lake from the eutrophic to hypereutrophic range (Fig. 8) and, therefore, if nothing else, *Chl*_BS_ retrievals provide an appropriate screening tool in familiar biogeophysical units.

Although coincident satellite-to-*in situ* match-ups analysis is the most common form of validation there are known challenges and sources of uncertainty associated with this approach (*e.g.*, Werdell and Bailey, 2005; Zheng and Di Giacomo, 2017; Neil et al., 2019). First, there is the issue of a single *in situ* sample representing an entire pixel (in our case, 300 m) that may or may not be homogenous. The potential intrapixel heterogeneity creates uncertainty about how well the *in situ* sample represents the mean across the larger pixel. And, as previously stated, multiple measurement techniques are often employed, all with their own uncertainties. Efforts to estimate the error associated with *Chl* laboratory techniques have found *in situ* sampling methods have an average error of 39%, and as high as 68% (Trees et al., 1985; Gregor and Maršálek, 2004). This is in the same range of the *Chl*_BS_ MAE_log_ of 1.6. Ideally, algorithm analysis would include an assessement of the uncertainties in field measurements as well. Unfortunately, the *in situ* data used in this study do not include uncertainty estimates, nor information on systematic, or directional, biases. Therefore, it is not possible to fully assess what amount of uncertainty results from the *in situ* data *versus* the algorithm itself.Another source of error for algorithms can be tied to diverse phytoplankton communities. Binding et al. (2019) found MCI and CI tended to underestimate *Chl* in diatom-dominated stations and performed best with cyanobacteria populations, particularly *Microcystis*-dominated waters, showing algorithm sensitivity to community composition. Ideally, the influence of community composition would be considered during algorithm development. The approach used in our analysis did not separate validation points by algal type as the information was not available in our *in situ* data set and, perhaps, some spread and error in the retrievals can be attributed to diversity in the phytoplankton populations considered. However, the *Chl*_BS_ algorithm is specifically for cyanobacteria dominated lakes, that is, because the algorithm is based on CI_cyano_, the process inherently filters for cyanobacteria dominated bodies of water. Nonetheless, mixed phytoplankton communities may register a valid CI_cyano_ value, which could introduce more error and uncertainty into the algorithm application. A future viable alternative approach may utilize validation that considers community composition and then applies a community-specific algorithm used to estimate *Chl*.

The bootstrapping approach using a CONUS match-up data set to modify the CI_cyano_ to *Chl* relationship allowed for coefficient adjustment and derivation of confidence intervals that resulted in an algorithm more suitable for the diverse set of CONUS lakes, which ranged from oligotrophic to hypereutrophic conditions. The *Chl*_BS_ exercise demonstrated that bootstrapping provides a useful alternative approach for remote sensing algorithm creation compared to the traditional least squares assignment of a linear relationship through the calibration points. Bootstrapping has been used successfully for *Chl* algorithms in other systems including in the Red Sea (Brewin et al., 2015) and the Great Lakes (Lesht et al., 2016), and offers an approach for consideration when using limited local data sets to develop a better performing algorithm for regional waterbodies. Another bootstrapping advantage is confidence intervals around the coefficients, which provide insights into the strength of the estimated relationships between the variables Fig. 8; C,D). Large confidence intervals, which could result from outliers in the data set, indicates a weak, poorly constrained relationship, while reduced confidence intervals would suggest a stronger algorithm. The approach made it possible to get relatively good estimates for CONUS, but for increased precision some regional tuning may be necessary.

## 5. Conclusions

We produced the first full standardized MERIS (2002–2012) inland water time series, inclusive of radiometric products and an indicator of cyanoHABs, CI_cyano_, for use universally in algorithm development and performance assessment, as well as CONUS plus Alaska water quality monitoring activities. The primary contribution of this work is the public distribution of this data set, with similar Sentinel-3A and −3B OLCI data sets planned to follow (see Appendix B). We also explored the derivation and utility of a CI_cyano_-to-*Chl* algorithm for application across CONUS. The estimation of *Chl* for inland waterbodies utilizing the readily available CI_cyano_ variable showed some potential. The original CI_cyano_-based *Chl* algorithm, *Chl*_T16_, was developed for eutrophic waters in Florida, USA (Tomlinson et al., 2016). A bootstrapping recalibration of the algorithm, *Chl*_BS_, demonstrated that the re-tuned algorithm is also appropriate for low *Chl* waters, albeit less precise and accurate in this range that for eutrophic conditions. Bootstrapping was demonstrated to be an effective approach to improve algorithm performance as large *in situ* data sets become available.

Ultimately, the need for reliable satellite remote sensing of inland bodies of waters for monitoring, management decisions, and global climate modeling has been well documented. A standardized and easily available satellite data set should substantially facilitate and enable future work in this arena. In addition, well-validated satellite algorithms can be used to create a historical inland waters data set that allows evaluation of changes in waterbodies over time. The *Chl*_BS_ algorithm explored in this work performed well for categorizing lakes into trophic status. These approaches are immediately available to support resource management decisions, such as cyanoHAB warnings, early detection activities, and lake trophic classification. However, the retrieval errors are relatively large and therefore may be unsuitable for precise estimates of *Chl*, therefore limiting the type of analyses that can be robustly interpreted. To that end, we offer a bootstrapping case study that provides confidence intervals as a step forward in uncertainty assessment. Perhaps more importantly, the contribution of ILW – a daily time series of more than 2000 lakes across CONUS and 5000 in Alaska–can further support community progress in the development and performance assessment of improved algorithms and approaches.

## Supplementary Material

Supplement1

## Figures and Tables

**Table 1:** *Chl*_T16_ and *Chl*_BS_ summary statistics (Bias_log_ and MAE_log_ in μg L^−1^) for total performance across 6 data ranges determined by *in situ Chl* concentrations using the evaluation data set (20% of the total data set). *Chl*_T16_ was developed for hypereutrophic lakes and is most appropriate for high *Chl* (>20 μg L^−1^) conditions; therefore, the 20–700 μg L^−1^ range results are shown.

*Chl* Concentration (μg L^−1^)	Statistics	*Chl*_*T16*_ (Tomlinson et al., 2016)	*Chl*_BS_
		Chl = 4050(±271) * CI_Cyano_ + 20 (±3)	Chl = 6620(±646) * CI_Cyano_ − 3.07(±5)

0–700	N	348	348
	Bias_log_	1.33	1.11
	MAE_log_	1.8	1.6
20–700	N	270	270
	Bias_log_	1.01	1.04
	MAE_log_	1.48	1.52
Oligotrophic/Mesotrophic	N	19	19
0–7	Bias_log_	8.2	1.79
	MAE_log_	8.2	2.8
Eutrophic	N	94	94
7–30	Bias_log_	2.23	1.27
	MAE_log_	2.2	1.8
Hypereutrophic	N	145	145
30–90	Bias_log_	1.16	1.19
	MAE_log_	1.3	1.4
High Hypereutrophic	N	82	82
>90	Bias_log_	0.60	0.73
	MAE_log_	1.7	1.5
