# Supplementary material for: Satellites for long-term monitoring of inland U.S. lakes: The MERIS time series and application for chlorophyll-a
Source: Remote Sens Environ. Author manuscript; Available in PMC 2022 Dec 1. (PMC9680834; doi:10.1016/j.rse.2021.112685)
Supplement: Supplement1 [file NIHMS1746942-supplement-Supplement1.docx]

**Appendix A: Acronyms and abbreviations**

Table A1. Acronyms, abbreviations, and symbols used throughout the paper

| Phrase | Acronym, Abbreivation, Symbol |
| --- | --- |
| chlorophyll-a | *Chl*; μg L^-1^ |
| Chlorophyll-a bootstrapped algorithm; based on Cyanobacteria Index-to-chlorophyll relationship | *Chl*_BS_ |
| Contiguous United States | CONUS |
| Cyanobacteria Assessment Network | CyAN |
| Cyanobacteria Harmful Algal Bloom | CyanoHAB |
| Cyanobacteria Index (Lunetta et al., 2015) | CI_cyano_; unitless |
| Cyanobacteria Index (Wynne et al., 2008) | CI; unitless |
| European Space Agency | ESA |
| Inland Waters Data Set | ILW |
| Mean Absolute Error calculated from log_10_ analysis | MAE_log_ |
| MEdium Resolution Imaging Spectrometer | MERIS |
| NASA Ocean Biology Processing Group | OBPG |
| Ocean and Land Colour Instruments | OLCI |
| Rayleigh-corrected top-of-atmosphere reflectance; rho_s | *ρ*_s_(λ); unitless |
| Spectral Shape | SS |
| Standard Mapped Images | SMI |

**Appendix B: Preliminary results for OLCI**

Reproductions of Figure 2 for OLCI on Sentinel-3A and -3B.


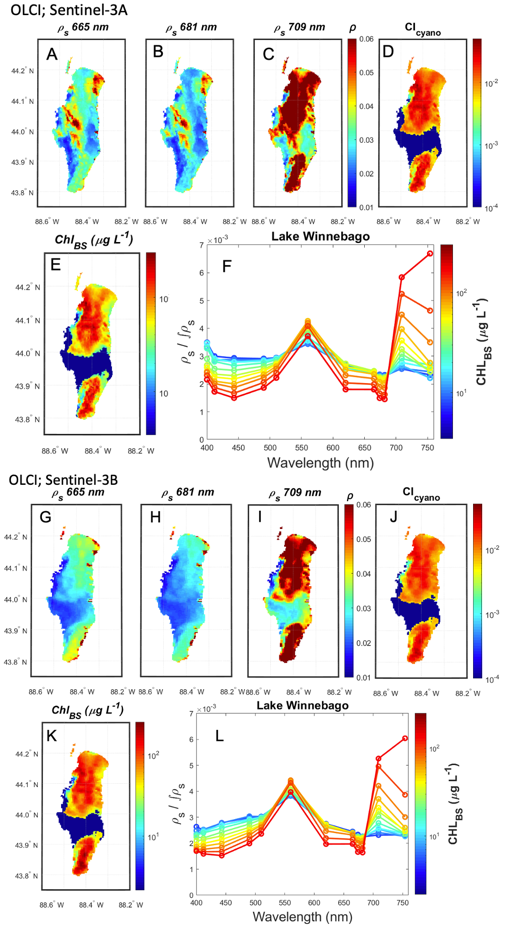


**Figure A1.** Example OLCI Sentinel-3A (top; A-F) and Sentinel-3B (bottom; G-L) satellite data from Lake Winnebago, Wisconsin from 13 July 2020 (A-F) showing mapped satellite imagery *ρ*_s_ (665, 681, 709 nm; A-C, G-I), CI_cyano_ (D,J) and *Chl*_BS_ (E,L) and *ρ*_s_(λ) spectra (F,L). The maps of each CI_cyano_ values and corresponding *Chl*_BS_ demonstrate different water types present (D-E;J-K). The median MERIS *ρ*_s_(λ) spectra are shown for pixels in discrete *Chl* ranges with colors representing diverse water types based on discretized ranges of *Chl* values (F,L). To focus on the variations in spectral shape, and not spectral amplitude, each *ρ*_s_ spectrum was normalized by its integrated value [*ρ*_s_(λ) / ∫*ρ*_s_ ] over the range of 400–754 nm. These spectra are processed as part of ILW. The circles along the lines mark the center wavelength of measured satellite bands (F,L). OLCI data is currently unavailable but will be added to the inland waters data set once quality control review is complete.

**
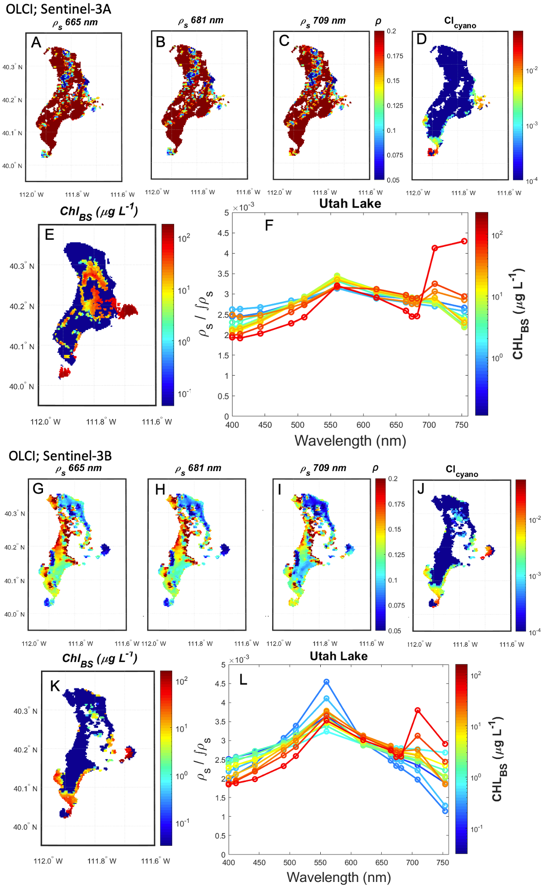
**

**Figure A2.** Example OLCI Sentinel-3A (top; A-F) and Sentinel-3B (bottom; G-L) satellite data from Utah Lake, Utah from 13 July 2020). White in the mapped lake area represents missing data due to clouds. Caption Figure A1 provides further details.

**Appendix C: Manipulation of the ILW dataset**

**Figure A3.** Map of the CyAN CONUS tiles. Blue represents CyAN resolvable lakes.

The ILW Standard Mapped Images (SMIs) can easily be extracted into smaller regions, reprojected from Plate Carrée into another map projection, and exported from netCDF into an alternate file format using the *l3mapgen* software packaged as part of the SeaWiFS Data Analysis System (SeaDAS; <https://seadas.gsfc.nasa.gov>). As an example, the following provides a recipe for using *l3mapgen* to recreate operational CyAN imagery. NASA provides operational CyAN imagery to the U.S. Environmental Protection Agency with the following architecture:

- Full CONUS is divided into 54 unique tiles (Figure A3),
- These data use an Albers conic projection, and
- The tiles are GeoTIFFS.

*l3mapgen* is available from a terminal command line or through the SeaDAS graphic user interface (GUI) that ultimately calls the same command line program. It operates using a series of input arguments, a full list for which exceeds the needs of this Appendix. Running *l3mapgen* from a terminal window without arguments will provide the full list of options. For this simple case study, the focus will be the three key arguments that reproject an input SMI into Albers conic, extract the tile 7_5 for Florida, USA (Figure A3), and export the data as a GeoTIFF. The bounding north, south, east and west latitude and longitude boundaries for tile 7_5 are 30, 25, -80, and -85, respectively. The arguments of interest are:

- “ifile” to identify the input SMI (or Level-3 bin) file,
- “ofile” to assign the output file name,
- “oformat” to define the output file format,
- “resolution” to retain the 300 m SMI spatial resolution,
- “projection” to assign the new map projection,
- “north” to assign the northernmost latitude for the extracted file,
- “south” to assign the southernmost latitude for the extracted file,
- “east” to assign the easternmost longitude for the extracted file, and
- “west” to assign the westernmost longitude for the extracted file,

The terminal calling sequence for this Florida tile case study is:

l3mapgen ifile=[intput file] ofile=[output file] oformat=tiff resolution=300.0 projection=albersconic north=30 south=25 east=-80 west=-85

Naturally, all of these arguments are also provided in the associated SeaDAS GUI. The resultant output file should mimic the operational CyAN data products generated by NASA.

The user will need to update the latitude and longitude boundaries to the bounds of their interest.

Below are options for output format (oformat) options that would assist in reprojecting the ILW SMI format.

            ....

netcdf4: netCDF4 file, can contain more than one product

       hdf4:    HDF4 file (old SMI format)

           png:     PNG image file

            ppm:     PPM image file

          tiff:    TIFF file with georeference tags

Further assistant and information is available at the SeaDAS site (<https://seadas.gsfc.nasa.gov>). Additionally, the Ocean Color Forum hosts active SeaDAS conversations sharing information and answering users questions (https://forum.earthdata.nasa.gov/app.php/tag/OBDAAC/AND?).
